# 

**Published:** 1870-01

**Authors:** 


### INDEX OF HALL'S JOURNAL OF HEALTH.

VOL. XVII., 1870.

| | PAGE. |
|---|---|
| Air we Breathe | 9 |
| Apoplexy | 194, 199 |
| Against Me | 223 |
| Asthma | 261 |
| Brandy as Medicine | 11 |
| Bread, Good | 235, 57 |
| Boarding Schools Girl | 67 |
| Bible | 227, 183 |
| Bad Blood | 99 |
| Broken Limbs | 213 |
| Bathing | 213 |
| Consumption | 249, 183, 35 |
| Cold, A Common | 49 |
| Corns | 100 |
| Church-Going | 120 |
| Corsets | 146 |
| Children's Studies | 176 |
| Children's Sleep | 200 |
| Cool Rooms | 201 |
| Cranberries | 210 |
| Country Physicians | 224 |
| Choked to Death | 232 |
| Dyspepsia | 1 |
| Drunkards, Terrible | 23 |
| Domestic Crime | 44 |
| Divorce | 45 |
| Dog's Love | 82 |
| Death of Dickens | 171 |
| Driven to Death | 254 |
| Doctors Dying | 257 |
| Eating, How Much | 760 |
| Eating, Object of | 62 |
| Early Rising | 105 |
| Exercise | 5, 116 |
| Eggs | 125 |
| Erect Gait | 127 |
| Eating Care | 217 |
| Failures in Business | 31 |
| Furnace Heat | 17, 42 |
| Franklin, Benjamin | 145 |
| Fruit Syrups | 176 |
| Fat Persons | 189 |
| Fainting | 194 |
| Fish and Meat | 234 |
| Fever and Ague | 244 |
| Fatuity | 263 |
| Gastric Juice | 3 |
| German Correspondence | 21 |
| Giving made Easy | 119 |
| German, Travel in | 121 |
| Guide Board | 242, 123 |
| German Literature | 179 |
| Germany, Travel in | 204 |
| Health Readings | 75 |
| Health by Good Living | 93 |
| Habit, Disposition of | 126 |
| Health and Fun | 209 |
| Hydrophobia | 214 |
| Hair Dye | 234 |
| Integrity | 18 |
| Infants, Care of | 202 |
| Insanity | 229 |
| Indellible Ink | 262 |
| Life and Death | 10 |
| Lead Poison | 265, 30 |
| Ladies and Babies | 53 |
| Luncheons | 65 |
| Life Insurance | 103 |
| Lean, To Get | 79 |
| Lawyer | 187 |
| Lead Colic | 265 |
| Milk, Pure | 10, 57 |
| Mind, The | 136 |
| Memories | 137 |
| Mosquitos | 195 |
| Madman | 196 |
| Meat and Men | 198 |
| Mad Stone | 216 |
| Miasm | 244 |
| Meat, Diseased | 263 |
| Never Safe | 25 |
| Nose-Bleed | 74 |
| Newspaper Cures | 80 |
| Night-Mare | 177 |

| | PAGE. |
|---|---|
| New Food | 267 |
| Paste | 12 |
| Popular Fallacies | 28, 101 |
| Passionate Punishment | 181 |
| Parks | 192 |
| Poison | 259 |
| Recreation | 15 |
| Rest for the Weary | 143 |
| School, Death in | 176, 13 |
| Sunshine | 14 |
| Smoky Chimneys | 17 |
| Sunny Side | 46 |
| Sleep | 194, 70 |
| Student Life, German | 63 |
| Singing in Church | 106 |
| Shower Baths | 117 |
| Sitting Erect | 120 |
| Stars of Night | 129 |
| Summer Rules | 177 |
| Sunday Rest | 183 |
| Summer Complaint | 190 |
| Seeds of Death | 207 |
| Sewing Machines | 238 |
| Stimulants | 252 |
| Social Intercourse | 259 |
| Stammering | 260 |
| Teacher, Wise | 16 |
| Tea and Coffee | 255, 138, 38 |
| Tight Lacing | 102 |
| Taking Cold | 77 |
| Tape Worms, (and in July No.) | 202 |
| Table Manners | 188 |
| Tongue | 236 |
| Teeth | 239 |
| Vain Hopes | 140 |
| Vegetarians | 148 |
| Ventilating Chambers | 256, 193 |
| Vaccination | 233 |
| Weather | 15 |
| Water on the Stove | 17 |
| Work and Wealth | 134 |
| Work Following | 180 |
| Wet Feet | 210 |
| Which is Right? | 211 |
| Woman's Helper | 240 |
| Whisky Cure | 258 |

